# High-frequency repetitive transcranial magnetic stimulation of the left dorsolateral prefrontal cortex may reduce impulsivity in patients with methamphetamine use disorders: A pilot study

**DOI:** 10.3389/fnhum.2022.858465

**Published:** 2022-08-05

**Authors:** Qingming Liu, Xingjun Xu, Huimin Cui, Lei Zhang, Zhiyong Zhao, Da Dong, Ying Shen

**Affiliations:** ^1^Center for Brain, Mind and Education, Shaoxing University, Shaoxing, China; ^2^Department of Psychology, Shaoxing University, Shaoxing, China; ^3^School of Psychology, Nanjing Normal University, Nanjing, China; ^4^Rehabilitation Medicine Center, The First Affiliated Hospital of Nanjing Medical University, Nanjing, China; ^5^School of Early-Childhood Education, Nanjing Xiaozhuang University, Nanjing, China; ^6^Key Laboratory for Biomedical Engineering of Ministry of Education, Department of Biomedical Engineering, College of Biomedical Engineering and Instrument Science, Zhejiang University, Hangzhou, China

**Keywords:** rTMS, left DLPFC, MAUD, craving, impulsivity

## Abstract

**Background:**

Individuals who use methamphetamine (MA) for a long period of time may experience decreased inhibition and increased impulsivity. In order to reduce impulsivity or improve inhibitory control ability, high-frequency repetitive transcranial magnetic stimulation (HF-rTMS) has attracted much attention of researchers. Recent studies on addiction have shown that rTMS can stimulate different brain regions to produce different therapeutic effects. Recent work also suggests that HF-rTMS over right dorsolateral prefrontal cortex (DLPFC) does not affect the impulsivity of patients with alcohol use disorder; while HF-rTMS over left DLPFC could improve the impulsivity of patients with alcohol use disorder and cigarette smokers. However, it should be noted that empirical studies applying HF-rTMS over left DLPFC of patients with MA use disorders (MAUD) (to evaluate its effect on impulsivity) are still lacking.

**Methods:**

Twenty-nine patients with MAUD underwent five sessions of HF-rTMS on the left DLPFC per week for 4 consecutive weeks. The cue-induced craving and stop-signal and NoGo task were assessed pre-rTMS and post-rTMS (at the end of the 4-week rTMS treatment). In addition, 29 healthy controls were recruited. There was no rTMS intervention for the controls, the performance of the stop-signal and NoGo task was evaluated on them.

**Results:**

In total, HF-rTMS of the left DLPFC significantly decreased MA-dependent patients’ cue-induced craving and stop-signal reaction time (SSRT). For SSRT, the pre-test of experimental group was significantly higher than the score of control group. In the experimental group, the pre-test score was significantly higher than the post-test score. For Go and stop-signal delay (SSD), the pre-test scores of the experimental group was significantly lower than the scores of the control group. No significant difference was found between the pre-test and the post-test scores of the experimental group.

**Conclusion:**

Add-on HF-rTMS of left DLPFC may be an effective intervention for reducing impulsivity and cue-induced craving of patients with MAUD. Future research with a control group of MAUD that does not undergo the treatment is needed to confirm the effectiveness.

## Introduction

Methamphetamine (MA) is a highly addictive and euphoric stimulant, which represents one of the largest illegal drugs in the world, and it has become more prevalent than other amphetamine derivatives (Jones et al., 2022). Among the registered drug abusers, there are approximately 1.35 million MA abusers, accounting for 56.1% of all drug abusers (People’s Republic of China Central Government | Ministry of Public Security, 2019). Generally, the use of amphetamine-like stimulants, including MA, is a major matter of public concern, representing the second most used substance after marijuana according to the United Nations Office on Drugs and Crime. It has been reported that repeated intake of MA would lead to drug addiction, the inability to control intake, strong drug craving, and the reduced prefrontal cortex function (Perry and Carroll, 2008; Koob and Volkow, 2010; Jentsch and Pennington, 2014; Sjoerds et al., 2014).

Impulsivity can be defined behaviorally as “actions, which are poorly conceived, prematurely expressed, unduly risky, or inappropriate to the situation, mainly resulting in undesirable consequences” (Dawe et al., 2004; Dalley and Robbins, 2017). The inability to stop an initiated response can be evaluated by a stop-signal task (SST) (Verbruggen and Logan, 2008). The task determines the time required between the Go signal and the stop signal for participants being capable of stopping the initiated response within a 50% probability range (i.e., stop-signal reaction time). That is to say, the higher the stop-signal reaction-time (SSRT), the more impulsive the individual is (Hamilton et al., 2015). Previous studies have shown that individuals with substance use disorders (including MA) exhibit an impaired performance on stop-signal impulsivity tasks (Lipszyc and Schachar, 2010; Smith et al., 2014). This may be attributed to the chronic damage of the substance to the dopaminergic and serotonergic prefrontal-subcortical networks, which is related to motor control (Volkow et al., 2001), and therefore, may affect performance on response inhibition task.

In order to reduce impulsivity or improve inhibitory control ability, transcranial magnetic stimulation (TMS) has attracted much attention of researchers (Bellamoli et al., 2014). TMS is a robust magnetic pulse generated by an electromagnetic coil, which can penetrate the skull and alter neural activity of the skull base tissue. Pulses delivered in series are called repetitive TMS (rTMS). Depending on the pulse frequency, either inhibitory [low-frequency (LF) ≤ 1 Hz] or excitatory [high-frequency (HF) ≥ 5 Hz] effects can be produced (Rossi et al., 2009; Guse et al., 2010). Effects of rTMS treatment include enhancing the release of dopamine in the limbic circuit of the brain and affecting the excitability of the brain nerves, ultimately lead to changes in neural adaptation (Strafella et al., 2001), continuous changes in cortical plasticity (Hallett, 2007), and reorganization of network functional connections (Philip et al., 2018). The dorsolateral prefrontal cortex (DLPFC) is frequently selected as the target region in inhibitory control networks (Ridderinkhof et al., 2004). Previous studies that applied HF-rTMS over DLPFC of patients with substance use disorders have obtained inconsistent results on impulsivity measures: for instance, it has been shown that while one-time frequency of 10 Hz does not improve accuracy on the Go-NoGo task (Herremans et al., 2013); quartic frequency of 10 Hz stimuli could improve accuracy on the Go-NoGo task (Del Felice et al., 2016) in alcohol-dependent patients. In nicotine-dependent patients, a single-frequency of 10 or 20 Hz could improve performance of a delay discounting task (Sheffer et al., 2013), which demonstrates that it could reduce impulsivity in patients with nicotine use disorders. Another study uses the 2-choice oddball paradigm, and the results show that low-frequency rTMS could attenuate the craving and impulsivity of patients with MAUD (Yuan et al., 2020). Up to now, no study has examined the effects of HF-rTMS on impulsivity in patients with MAUD, as measured by the stop-signal and NoGo task.

rTMS could reduce cue-induced craving in patients with MAUD (Liu et al., 2017, 2020b), and some studies have shown that craving is positively correlated with impulsivity (Mathew et al., 2015; Li et al., 2021). Recent work also suggests that after the first treatment, there is no change in craving of patients with MAUD, while after 5 HF-rTMS treatment, there is a significant reduction in craving; while sham stimulation does not have the same effect (Su et al., 2017). In addition to reducing cravings, it suggests that long-term HF rTMS can improve withdrawal symptoms, anxiety and depression scores, sleep quality and cognition in patients with MA or heroin use disorders (Su et al., 2017; Liang et al., 2018; Lin et al., 2019). Other studies have shown that HF-rTMS of the left DLPFC may be an effective intervention for the treatment of cocaine use disorder symptoms such as anhedonia and craving, which needs to be further explored in larger placebo-controlled studies (Terraneo et al., 2016; Pettorruso et al., 2018).

In the present study, we investigated the effects of HF-rTMS on impulsivity of patients with MAUD. We hypothesized that repeated add-on HF-rTMS of left DLPFC may be effective in improving impulse control ability in patients with MAUD, as well as reducing their cue-induced craving. Therefore, we proposed that patients with MAUD who received HF-rTMS would show no difference in impulsivity compared with healthy controls (HCs). In the present study, we recruited 29 patients with MAUD and 29 HCs. Patients with MAUD were assessed with the stop-signal and NoGo task 1 day before and 1 day after HF-rTMS treatment, and healthy controls were assessed at baseline.

## Methods

### Participants

**FIGURE 1:**
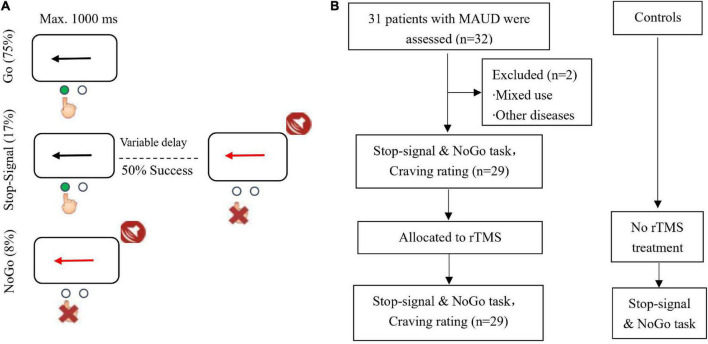
**(A)** Task and stimuli. **(B)** Study design. MAUD, methamphetamine use disorder.

A total of 29 male patients with MAUD, right-handed, and were admitted to an addiction rehabilitation center in Zhejiang Province (China), were recruited. Inclusion criteria were as follows:(1) patients who aged 18–65 years old; (2) patients who had a recurrent history of MAUD (DSM-V diagnosis, urine test was positive on admission, abstinence thereafter); (3) patients with a mild or higher level of drug craving; (4) patients who signed the written informed consent form; (5) patients who had not received TMS treatment at 6 months prior to the experiment; (6) patients who did not receive other therapeutic strategies, such as pharmacological or psychological treatment, etc., during the study. Exclusion criteria were as follows: (1) patients with neurological disorders; (2) patients with cardiovascular diseases; (3) patients with other serious physical diseases; (4) patients with psychiatric disorders; (5) patients with a history of brain injury; (6) patients with a history of epilepsy; (7) pacemaker wearers; (8) participants who, according to an investigator’s judgment, were not eligible or had a poor compliance. Withdrawal and termination criteria were as follows: (1) serious violations of the clinical trial protocol; (2) participants who could not follow the protocol for treatment and had a poor compliance; (3) intolerable adverse events; (4) subjects who voluntarily withdrew the study at any time. An investigator determined whether a participant needs to withdraw or continue the trial based on the above-mentioned criteria. Every effort must be made to complete the efficacy and safety checklists specified in the protocol at the time of withdrawal from the trial, and to fully document the reasons for withdrawal and adverse effects. An investigator attempted to suggest or provide new or alternative treatment methods to participants based on their clinical conditions. In addition, 29 male HCs without a history of major neurological or psychiatric diseases from a volunteer group in Brain and Cognitive Neuroscience at Liaoning Normal University (Dalian, China) were recruited, the two groups were matched for education and age. The study was approved by the Ethics Committee of Nanjing Normal University (Nanjing, China; Approval No. 2017-004) and was registered in the Chinese Clinical Trial Registration Center (no. ChiCTR17013610^1^), and the written informed consent forms were signed by all participants prior to beginning the study (Figure 1A).

### Assessment of cue-induced craving

For the assessment of cue-induced craving, participants watched a video about MA use for 5 min, the evaluation was performed as described previously (Liu et al., 2020b).

Participants were asked to express their desire to use drugs immediately and mark it on the visual analogue scale of 0–100. The question was scored as follows: What is the level of your craving for MA? (0 indicates absolutely unwilling and 100 represents extremely tendentious). Participants’ data were fully kept confidential and were not be provided to the judiciary, and the authenticity of videos was evaluated.

### Implementation of the HF-rTMS and study design

Consistent with previous study (Liu et al., 2020a), the stimulation protocol included 20 weekdays (once a day, 5 days on, 2 days off/week, 10 Hz frequency, pulse intensity 100% of the resting motor threshold, 5 s on, 10 s off, repeated for 40 times, 2,000 pulses) and targeted the left DLPFC, which was performed in this study. During the operation, the stimulus intensity was adjusted according to participants’ tolerability, single-pulse TMS was used to measure the motor threshold of the motor cortex, and it was adjusted until the response of thumb muscle was observed in 5 out of 10 stimuli. During the treatment, the coil was placed in the left prefrontal area, 5 cm away from the scalp area of the measured movement threshold (Liu et al., 2017, 2019). The HF-rTMS was applied on the left DLPFC using a CCY-IA TMS device (Yiruide Biotechnology Co., Ltd., Wuhan, China) and a circular coil was used.

Before commencing the study, an investigator screened MA addicts who were voluntarily participated, excluded those cases with contraindications to TMS, obtained their consent, and collected their basic data, including their full-name, age, years of drug use, maximum amount of drug consumption per session, maximum amount of use per month, and type of addiction (excluding mixed use of other drugs, such as heroin and marijuana). Then, an investigator assessed the craving scores of the experimental group and eliminated participants with craving scores of < 40. Twenty-nine patients with MAUD underwent five sessions of HF-rTMS on the left DLPFC per week for 4 consecutive weeks. The cue-induced craving and stop-signal and NoGo task were assessed pre-rTMS and post-rTMS (at the end of the 4-week rTMS treatment). However, HCs were not asked to receive TMS treatment and only the stop signal and NoGo task was conducted. During treatment, participants might experience discomfort or side effects. Therefore, we used a self-rating scale for patient-perceived side effects. The side effects included headache, neck pain, scalp pain, tingling sensation, itching, burning sensation, skin redness, sleepiness, lack of concentration and changes in mood, discomfort at the site of stimulation, etc. Each side effect was scored on a scale of 1–10. After each treatment, side effects were monitored.

Stop-signal and NoGo task: This task consisted of randomly interspersed NoGo and stop-signal trials with inclusion of 360 Go (75%), 40 NoGo (8%), and 80 stop-signal trials (17%). In the Go trials, participants responded to the black arrow (1000 ms) in the left and right directions by pressing a button with their right hand. Participants could respond to the questions with either the index finger (pressing the left arrow) or the middle finger (pressing the right arrow). In the stop-signal trials, the initial response was prompted by the left or right black arrow, while when the stop-signal was delayed, the arrow color changed to red and appeared simultaneously with the sound. At this time, participants were asked to avoid responding. The stop-signal delay (SSD) maintained a successful inhibition of 50% by using an ascending or descending algorithm with an initial estimation of 250 ms varying from trial to trial (Chamberlain et al., 2007). SSD indicates the time interval between response signal and stop signal. This is similar to the tracking algorithm used in a previous stop-signal task imaging study (Cubillo et al., 2014). In the NoGo task, participants were asked to avoid responding to the red arrow (1000 ms) and the accompanying beep, which would be equivalent to a 0 s SSD. SSRT indicates the time from the appearance of the stop signal to the completion of the stop task, i.e., the internal reaction time of the subject when successfully suppresses an action impulse. SSRT is the most important indicator in the stop signal task, responding to the subject’s reaction speed to the stop signal. Most studies have used it as a direct indicator of response inhibition ability to assess whether the subject has a deficit in response inhibition. A higher SSRT has been suggested to represent that participant has a longer stop-signal response time, a poorer behavioral inhibition, and a higher impulsivity. In contrast, a lower SSRT indicates that participants have a shorter stop signal response time, a better behavioral inhibition, and they can promptly inhibit impulsive behaviors. Participants were asked to operate with their right hand and ensure that they fully understood the cognitive task before performing it, so that they could respond as quickly as possible and ensure the accuracy. The participants were asked to remain quiet and not to interact with each other while waiting. They were asked to stay focused during the task. The stop-signal and NoGo task are shown in Figure 1B.

### Statistical analysis

Statistical analysis was performed using the SPSS 19.0 software (IBM Corp., Armonk, NY, United States). The independent-sample *t*-test was used to compare differences in demographic variables between the experimental and control groups. The independent-sample *t*-test was employed to compare differences in the performance of the stop-signal and NoGo task between the experimental group and the control group. The paired *t*-test was utilized to compare differences in stop-signal and NoGo task between the pre and post experimental group. Pearson correlation analysis was adopted to analyze the relationship between experiment variables (craving, SSRT, Go, SSD, etc.) and demographic variables. *p* < 0.05 was considered statistically significant.

## Results

### Participants’ demographic characteristics

The participants’ demographic characteristics are listed in Table 1, and no significant difference could be found in age and education between the experimental group and the control group (*M* ± SEM).

**TABLE 1:** Participants’ demographic characteristics (*M* ± *SEM*).

Variable	Experimental group	Control group	*t*	*P*
Sex	Male	Male	–	–
Age (years)	35.66 ± 1.49	36.55 ± 1.80	0.38	0.70
Education (years)	8.90 ± 0.39	9.69 ± 0.39	1.66	0.10
Duration of drug use (years)	8.86 ± 0.63	–	–	–
Maximum usage per session (g)	0.93 ± 0.09	–	–	–
Monthly usage (g)	16.31 ± 2.41	–	–	–

There was no significant difference in age [*t*(56) = 0.38, *p* = 0.70] and education [*t*(56) = 1.66, *p* = 0.10] between the experimental group and the control group.

### Comparison between the pre-test experimental group and the control group

The independent-sample *t*-test was employed to compare SSRT, Go, and SSD between the experimental group (pre-test) and the control group. For SSRT, the pre-test of experimental group (*M* = 285.53, SEM = 7.90) was significantly higher than the score of control group (*M* = 257.59, SEM = 6.71), *t*(56) = 2.38, *p* < 0.05. Therefore, it suggested that patients with long-term MAUD showed decreased inhibitory control ability. For Go, the pre-test of experimental group (*M* = 490.19, SEM = 16.48) was significantly lower than the baseline score of control group (*M* = 555.74, SEM = 17.06), *t*(56) = 3.31, *p* < 0.01. The Go reaction time of patients with MAUD was significantly lower than that of health control, suggesting that patients with MAUD were deficient in behavioral inhibition and showed more inhibition control disorders. This finding is consistent with previous research (Qi et al., 2022), which reports impaired inhibitory control in internet addiction disorder. For SSD, the pre-test of experimental group (*M* = 204.66, SEM = 20.42) was significantly lower than the baseline score of control group (*M* = 298.15, SEM = 17.18), *t*(56) = 3.68, *p* < 0.001.

### Comparison between pre- and post-test scores of the experimental group

The paired *t*-test was used to compare craving score between the experimental group of pre- (65.17 ± 3.20) and post-test (35.86 ± 3.20). It was revealed that HF-rTMS of the left DLPFC decreased craving score [*t*(28) = 7.33, *p* < 0.001] of patients with MAUD.

**FIGURE 2:**
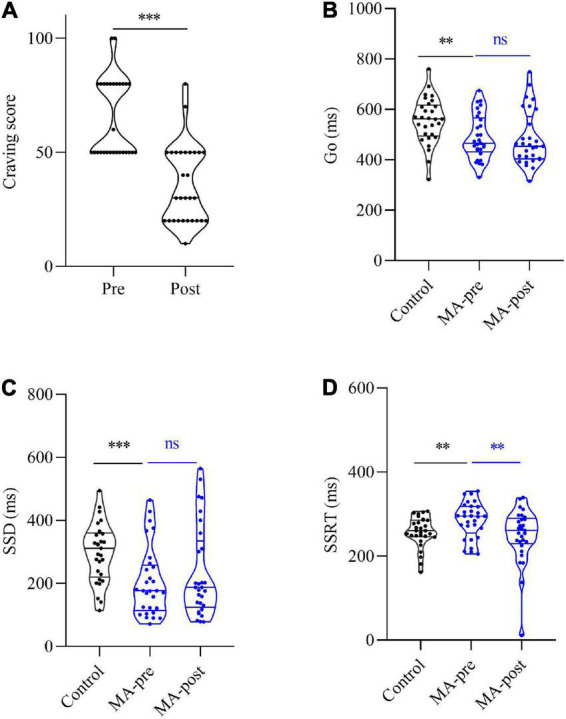
Comparison craving score between the experimental group of pre- and post-test. **(A)** The pre-test was significantly higher than the post-test score of the experimental group [*t*(28) = 7.33, *p* < 0.001] of patients with MAUD. Comparison of SST and NoGo between experimental and control groups. **(B)** For Go, the pre-test of the experimental group was significantly lower than the score of the control group [*t*(56) = 3.31, *p* < 0.01], and the post-test of the experimental group was also significantly lower than the score of the control group [*t* (56) = 3.21, *p* < 0.01]. No significant difference was found between the pre-test and the post-test scores of the experimental group [*t*(28) = 0.71, *p* = 0.49]. **(C)** For SSD, the pre-test of the experimental group was significantly lower than the score of the control group [*t*(56) = 3.68, *p* < 0.001], and the post-test of the experimental group was also significantly lower than the score of the control group [*t*(56) = 2.32, *p* < 0.05]. No significant difference was found between the pre-test and the post-test scores of the experimental group. **(D)** For SSRT, the pre-test of the experimental group was significantly higher than the score of the control group [*t*(56) = 2.38, *p* < 0.05]. There was no significant difference between the post-test of the experimental group and the score of the control group [*t*(56) = 0.54, *p* > 0.05]. The pre-test was significantly higher than the post-test score of the experimental group [*t*(28) = 2.77, *p* < 0.05]. [*t*(28) = −1.63, *p* = 0.12]. The symbol * represents *p* < 0.05, ** represents p < 0.01, and *** represents *p* < 0.001.

The paired *t*-test was utilized to compare the SSRT, Go, and SSD between the experimental group of pre-test and post-test. For SSRT, the pre-test experimental group (*M* = 285.53, SEM = 7.90) was significantly higher than the post-test experimental group (*M* = 249.62, SEM = 12.11), *t*(28) = 2.77, *p* < 0.05. This indicated that HF-rTMS intervention could reduce impulsivity and improve inhibitory control ability of patients with MAUD. For Go, there was no significant difference between the pre-test experimental group (*M* = 490.19, SEM = 16.48) and the post-test experimental group (*M* = 483.25, SEM = 20.17), *t*(28) = 0.71, *p* = 0.49. For SSD, there was no significant difference between the pre-test experimental group (*M* = 204.66, SEM = 20.42) and the post-test experimental group (*M* = 233.63, SEM = 27.03), *t*(28) = −1.63, *p* = 0.12, (Figure 2).

In conclusion, the HF-rTMS treatment had an effect on SSRT, but not on Go and SSD in the stop-signal and NoGo task of patients with MAUD.

### Correlation between test scores and demographic variables

**FIGURE 3:**
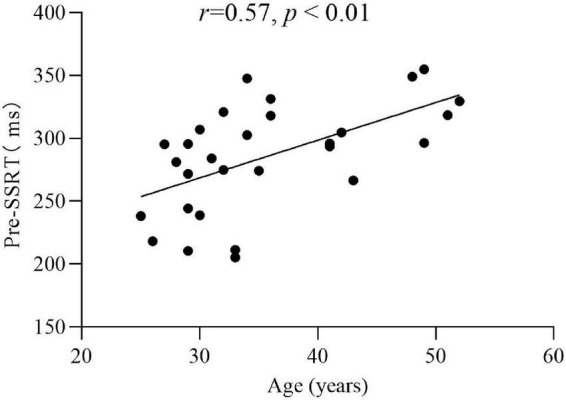
Correlation analysis of the pre-SSRT and age in the experimental group (*r* = 0.57, *p* < 0.01).

Pearson correlation analysis was used to analyze the correlation between SSRT, Go, SSD and demographic variables in the experimental group. The results showed that pre- SSRT was significantly positively correlated with age (*r* = 0.57, *p* < 0.01) (Figure 3). This indicated that the older the participants, the greater the SSRT; while post-SSRT (*r* = 0.03, *p* > 0.05), pre- (*r* = −0.08, *p* > 0.05), post-Go (*r* = −0.13, *p* > 0.05), pre- (*r* = −0.28, *p* > 0.05), and post-SSD (*r* = −0.11, *p* > 0.05), these variables were not significantly correlated with age. The correlation between pre-SSRT and years of drug use (*r* = −0.03, *p* > 0.05), maximum consumption per time (*r* = −0.06, *p* > 0.05), and monthly use (*r* = 0.14, *p* > 0.05), respectively, was not significant. The correlation between post-SSRT and years of drug use (*r* = 0.03, *p* > 0.05), maximum consumption pre time (*r* = −0.12, *p* > 0.05), and monthly use (*r* = −0.04, *p* > 0.05), respectively, was not significant. The pre- and post-test differences of SSRT, Go and SSD were not significantly correlated with any demographic variables (all *p* > 0.05).

### Safety and tolerability

In the experimental group, a total of 580 treatments were performed. There were 9 cases of mild headache after stimulation (1.6%) of 3 subjects, 2 reported 4 times and 1 reported 1 time, 6 cases of scalp pain (1.0%) of 2 subjects who reported 3 times respectively, 10 cases of sleepiness (1.7%) of 6 subjects, 4 reported 2 times and 2 reported 1 time, and 12 cases of discomfort after treatment (2.1%) of 9 subjects, 6 reported 1 time and 3 reported 2 times. Three subjects reported both headaches and discomfort after treatment. All of these discomforts disappeared in the follow-up 1 week later.

## Discussion

The present study aimed to investigate the effects of repeated HF-rTMS treatment on impulsivity in patients with MAUD. The HF-rTMS treatment was well tolerated and no serious side effects could be observed. The stop-signal and NoGo task was used to evaluate the impulsivity of patients with MAUD before and after HF-rTMS treatment. The results of the present study suggested that repeated HF-rTMS may be effective in reducing drug-induced craving in patients with MAUD, as well as improving their impulse control ability.

To our knowledge, this study is the first to assess the influences of HF-rTMS treatment on impulsivity in patients with MAUD, as measured by the stop-signal and NoGo task. One study explored the effects of HF-rTMS on impulsivity tasks in patients with alcohol use disorder using three tasks simultaneously—SST, Go-NoGo and delay discounting task. It was found that HF-rTMS did not improve alcohol use disorder patients’ performance on impulsivity tasks (Schluter et al., 2019), and participants in the study only received 10 treatments; whereas participants in our study received five sessions of HF-rTMS on the left DLPFC per week for 4 consecutive weeks of the therapy. It might be also due to the different clinical statuses of treated participants (i.e., a poorer clinical status requires more stimulation to achieve a greater efficacy).

We will briefly outline the limitations of this study. First, our study was the absence of a sham control group receiving routine rehabilitation treatment without rTMS intervention. The patients of MAUD did not use MA during rTMS treatment, but they did normal rehabilitation exercises. Thus, the results observed in the patients of MA group could not be solely attributed to the unique effects of rTMS. Second, the study lacked associated impulsivity scales to measure impulsivity in patients with MAUD; in the future studies, some impulsivity scales should be added, such as the Barratt Impulsiveness Scale Version 11 (BIS-11), of which is able to assess motor impulsivity, cognitive impulsivity, and unplanned impulsivity of participants. Third, to some extent, in the absence of a sham control group, the placebo effect may still exist in the experiment group, the experiment perhaps is only a pilot study at present. Finally, as participants in this study were all in the same brigade in a rehabilitation center, they might exchange their physical feelings with each other during the HF-rTMS treatment.

The current study suggested that HF-rTMS might reduce impulsivity and craving of patients with MAUD. It was also found in previous research that HF-rTMS could reduce the craving of patients with heroin usage (Shen et al., 2016). However, the underlying neurophysiological mechanism has remained elusive. Psychostimulants has the potential to cause changes in the prefrontal cortex network, thereby increasing impulsive behavior (Volkow et al., 2001; Badiani et al., 2011; Ersche et al., 2011); while HF-rTMS could facilitate cortical excitability (Peterchev et al., 2012), thus improve the inhibitory control ability of addicts. In addition, it is highly crucial to indicate whether intermittent theta-burst stimulation could achieve the same effect. Thus, further studies need to be carried out to eliminate the above-mentioned deficiencies and to confirm our findings.

## Conclusion

The research revealed that add-on HF-rTMS of left DLPFC might be an effective intervention for reducing impulsivity and cue-induced craving of patients with MAUD. Furthermore, future studies are required to explore the underlying cognitive and neural mechanisms.

## Data availability statement

The raw data supporting the conclusions of this article will be made available by the authors, without undue reservation.

## Ethics statement

The study was approved by the Ethics Committee of Nanjing Normal University (Nanjing, China; Approval No. 2017-004) and was registered in the Chinese Clinical Trial Registration Center (http://www.chictr.org.cn; no. ChiCTR17013610), and the written informed consent forms were signed by all participants prior to beginning the study.

## Author contributions

QL, DD, and YS: conceptualization. YS: funding acquisition and supervision. QL and XX: investigation and writing–original draft. QL and DD: methodology. QL: project administration. QL, HC, LZ, ZZ, and DD: writing–review and editing. All authors have read and agreed to the published version of the manuscript.

## Footnotes

1
http://www.chictr.org.cn
